# gdGSE: An algorithm to evaluate pathway enrichment by discretizing gene expression values

**DOI:** 10.1016/j.csbj.2025.04.038

**Published:** 2025-05-01

**Authors:** Jiangti Luo, Qiqi Lu, Mengjiao He, Xiaobo Zhang, Xiang Yang, Xiaosheng Wang

**Affiliations:** a Biomedical Informatics Research Lab, School of Basic Medicine and Clinical Pharmacy, China Pharmaceutical University, Nanjing 211198, China; b Intelligent Pharmacy Interdisciplinary Research Center, China Pharmaceutical University, Nanjing 211198, China; c Big Data Research Institute, China Pharmaceutical University, Nanjing 211198, China; d Institute of Innovative Drug Discovery and Development, China Pharmaceutical University, Nanjing 211198, China; e School of Science, China Pharmaceutical University, Nanjing 211198, China; f Jiangsu Key Laboratory of Carcinogenesis and Intervention, School of Basic Medicine and Clinical Pharmacy, China Pharmaceutical University, Nanjing 210009, China; g Department of Oncology, JunXie Hospital, Nanjing University of Chinese Medicine, Nanjing 210002, China

**Keywords:** Gene set enrichment analysis, Discretization of gene expressions, Bulk and single-cell transcriptomes, Cancer subtyping, Cell type identification

## Abstract

We proposed gdGSE, a novel computational framework for gene set enrichment analysis. Unlike conventional methods that rely on continuous gene expression values, gdGSE employs discretized gene expression profiles to assess pathway activity. This approach effectively mitigates discrepancies caused by data distributions. This algorithm consists of two steps: (1) applying statistical thresholds binarizing gene expression matrix, and (2) converting the binarized gene expression matrix into a gene set enrichment matrix. Our results demonstrated that gdGSE could robustly extract biological insights from a diverse array of simulated and real bulk or single-cell gene expression datasets. Notably, gene set enrichment scores by gdGSE exhibited enhanced utility in downstream applications: (1) precise quantification of cancer stemness with significant prognostic relevance; (2) enhanced clustering performance in stratifying tumor subtypes with distinct prognoses; and (3) more accurate identification of cell types. Remarkably, the pathway activity scores by gdGSE showed > 90 % concordance with experimentally validated drug mechanisms in patients-derived xenografts and estrogen receptor-positive breast cancer cell lines. Our algorithm proposes that discretizing gene expression values provides an alternative method for evaluating pathway enrichment, applicable to both bulk and single-cell data analysis.

## Graphical Abstract

**Figure:**
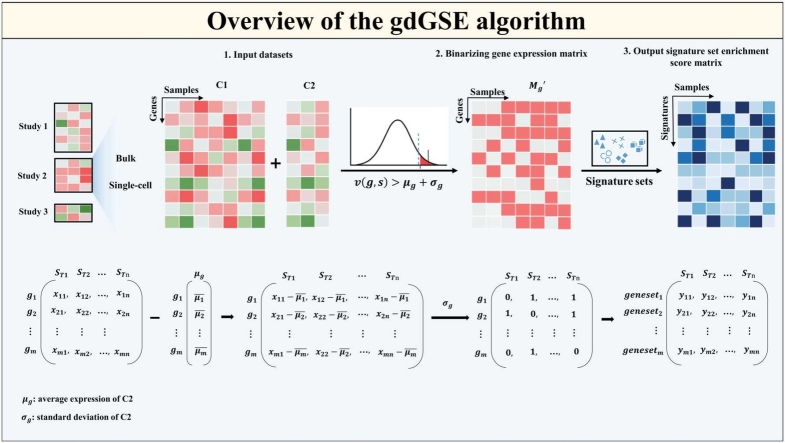

## 1 Introduction

Based on the transcriptomic profile of a set of genes, gene set enrichment analysis (GSEA) evaluates the enrichment levels of predefined gene modules to represent biological pathway activities or processes [1]. GSEA has emerged as a commonly-used method in downstream analyses of bulk and single-cell transcriptomic data, including disease subtyping [2], [3], gene signature scoring [4], [5], prognostic biomarker discovery [6]. GSEA offers two key advantages over single-gene analyses: (1) GSEA may overcome the outliers and dropouts in individual genes’ expressions through integrating multiple gene expression values into a single value; and (2) it can display biological findings in a more straightforward and explainable manner by pathway or signature enrichment characterization. To this end, numerous GSEA algorithms have been developed for downstream analysis of transcriptomic data. For bulk transcriptomes, methods such as ssGSEA (single-sample Gene Set Enrichment Analysis) [4] and PLAGE (Pathway-Level Analysis of Gene Expression) [7] leverage gene rank normalization and matrix factorization, respectively. For single-cell transcriptomes, the established pathway enrichment evaluation tools included AUCell [5], AddModuleScore [8], Pagoda2 [9], and singscore [10]. Despite these advancements, existing methods predominantly rely on continuous gene expression values, which are susceptible to batch effects and distributional biases across experimental conditions. This limitation underscores the need for robust frameworks that explicitly account for condition-specific expression dynamics while preserving biological interpretability.

A hallmark distinction between single-cell and bulk transcriptomes lies in the prevalence of dropout events—the frequent undetected expression of genes in single-cell samples. These dropouts may introduce systematic bias in downstream analysis, such as differential expression analysis, clustering, and cell trajectory inference. Because single-cell and bulk transcriptomes exhibit different data distribution, the GSEA methods originally developed for bulk data may not directly applicable to single-cell data, and vice versa [11]. For instance, the ssGSEA algorithm designed for bulk transcriptomes relies on gene ranks; while these ranks are largely continuous in bulk expression data, they are disrupted in single cell data due to dropouts. Noureen et al. [11] have demonstrated that ssGSEA’s susceptibility to rank distortion by dropouts may lead to erratic pathway scores, undermining its reliability in single-cell contexts. AUCell proposed for single-cell transcriptomes intuitively addresses dropout effects by focusing on top highly expressed genes, which is not propitious for bulk data with few dropouts. Furthermore, the gene ranks-based algorithms like ssGSEA and AUCell inherently compare gene expression levels within samples, disregarding baseline expression differences between genes (e.g., housekeeping genes versus lowly expressed regulators). This oversight may lead to the conflation of technical artifacts with biological signals, particularly in cross-condition comparisons (e.g., tumor versus normal samples).

Here we present a novel GSEA method, termed gdGSE, a threshold-based GSEA framework that leverages discretized gene expression profiles to address distributional discrepancies between bulk and single-cell transcriptomes. Additionally, gdGSE adjusts for the inherent baseline expression differences among different genes by normalizing with the reference gene expressions. Applying the algorithm to analyze a wide array of transcriptomic data, including simulated and real bulk or single-cell mRNA expression datasets, we demonstrated the universal applicability and robustness of gdGSE. Furthermore, compared with ssGSEA, PLAGE, z-score [12], AUCell, UCell [13], AddModuleScore, singscore, and pagoda2, gdGSE showed certain advantages in investigations of disease subtyping, cell type identification, drug responses and prognostic relevance.

## 2 Methods

### 2.1 gdGSE algorithm

The gdGSE algorithm takes bulk or single-cell transcriptomes as input, supposing that the bulk or single-cell samples have two different conditions, termed *C1* and *C2*, such as *C1* = “tumor” and *C2* = “normal”. The enrichment score of a gene set in a sample produced by gdGSE is a score in the sample’s condition relative to the other condition, such as tumor versus normal samples. The gdGSE methodology comprises two main steps:

Step 1: Binarizing gene expression values

For a gene expression matrix $M_{g}$ containing samples from two conditions (*C1* and *C2*), the expression value v(g,s) of gene g in sample s (where s∈ *C1*) is binarized based on the statistical properties of g′s expressions in samples from condition *C2*. The binarized gene expression matrix $M_{g}\prime$ is defined as:

$M_{g}^{\prime }\left(g,s\right)=\left\{\begin{array}{c} 1,\&\mathrm{if}v\left(g,s\right)\;>\mu_{g}+\sigma_{g},\\ 0,\&\mathrm{otherwise}, \end{array}\right)$

where $\mu_{g}=\;\frac{1}{\mathrm{|C}2|}\sum_{s^{\prime }\in C2}v(g,s\prime )$ (mean expression value of gene g in all *C2* samples), and $\sigma_{g}=\sqrt{\frac{1}{\mathrm{|C}2|}\sum_{s^{\prime }\in C2}{\left(v\left(g,s^{\prime }\right)-\mu_{g}\right)}^{2}}$ (standard deviation of g′s expression in all *C2* samples). Thus, the entries in $M_{g}\prime$ are 0 or 1.

Step 2: Calculating gene set enrichment scores

For a given gene set GS (containing G genes), its enrichment score (ES) in sample s∈C1 is computed as: $\mathrm{ES}\left(s,\mathrm{GS}\right)=\frac{G_{S}}{G}$, where $G_{S}=\sum_{g\in \mathrm{GS}}M_{g}^{\prime }\left(g,s\right)\;$(number of genes in GS with binarized value 1 in sample s, and G=|GS| (total number of genes in the gene set GS. Thus, we obtain a gene set enrichment score matrix, where each entry represents the enrichment of GS in a sample in condition C1 relative to C2.

**Fig. 1:**
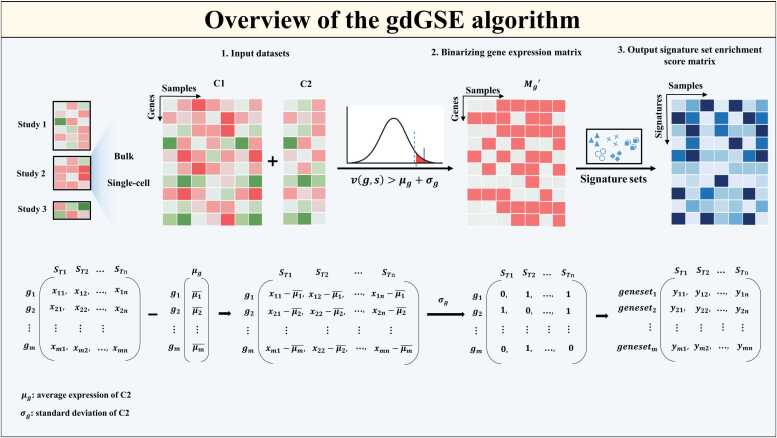
Overview of the gdGSE algorithm.

An overview of the gdGSE algorithm is illustrated in Fig. 1. An R package has been developed to implement the algorithm, which is available at https://github.com/WangX-Lab/gdGSE.

### 2.2 Data acquisition and preprocessing

All data here used for analysis are publicly available. We downloaded cancer genomics datasets for 28 TCGA cancer types from the UCSC Xena project (https://xenabrowser.net/datapages/). The 28 cancer types included adrenocortical carcinoma (ACC), bladder urothelial carcinoma (BLCA), breast invasive carcinoma (BRCA), cervical squamous cell carcinoma and endocervical adenocarcinoma (CESC), cholangiocarcinoma (CHOL), colon Adenocarcinoma and rectum adenocarcinoma (CRC), lymphoid neoplasm diffuse large B-cell lymphoma (DLBC), esophageal carcinoma (ESCA), glioma (low-grade glioma and glioblastoma multiforme), head and neck squamous cell carcinoma (HNSC), kidney chromophobe (KICH), kidney renal clear cell carcinoma (KIRC), kidney renal papillary cell carcinoma (KIRP), liver hepatocellular carcinoma (LIHC), lung adenocarcinoma (LUAD), lung squamous cell carcinoma (LUSC), ovarian serous cystadenocarcinoma (OV), pancreatic adenocarcinoma (PAAD), pheochromocytoma and paraganglioma (PCPG), prostate adenocarcinoma (PRAD), sarcoma (SARC), skin cutaneous melanoma (SKCM), stomach adenocarcinoma (STAD), testicular germ cell tumors (TGCT), thyroid carcinoma (THCA), thymoma (THYM), uterine corpus endometrial carcinoma (UCEC), uterine carcinosarcoma (UCS), and TCGA pan-cancer cohort. For each cancer type, we obtained its gene expression profiling (RNA-seq, RSEM normalized) and survival data. Because five cancer types (ACC, CESC, OV, SKCM, and TGCT) contained no normal control samples, we used the combined TCGA and GTEx gene expression (RSEM normalized) data from the UCSC Xena project. We downloaded single-cell RNA-seq (scRNA-seq) datasets from the NCBI Gene Expression Omnibus (GEO) (https://www.ncbi.nlm.nih.gov/geo/), including: (1) GSE113922 for pancreatic cancer tumors patient-derived xenograft (PDX) [14]; (2) GSE153509 for breast cancer cell line [15]; (3) GSE221553 for melanoma [16]; (4) GSE173706 for human normal skin [17]; (5) GSE111672 for pancreatic ductal adenocarcinoma (PDAC) [18], containing scRNA-seq (PDAC_SC) and spatial transcriptomic data (PDAC_ST); and (6) GSE149614 for hepatocellular carcinoma (HCC) [19]. For all these scRNA-seq datasets, we performed quality control of reads and cells following the quality control steps described in the original publications. For cell type annotation, we adopted original methods provided by these datasets-associated publications as ground truth. A summary of these datasets is presented in Supplementary Table S1.

### 2.3 Data simulation

Pseudobulk expression profiles were generated by aggregating raw gene expression counts from single cells within each simulated sample, emulating bulk-level resolution while preserving cell-type heterogeneity. For scRNA-seq data simulations, we used a deep learning-based spatial deconvolution algorithm, Bulk2Space [20], to generate spatially resolved single-cell expression profiles. To be specific, we used a scRNA-seq dataset and a spatial transcriptomic dataset for a PAAD patient (PDAC-A) [18] as the reference to deconvolute the gene expression profiles in four PAAD patients and a normal tissue in the TCGA-PAAD dataset. This analysis produced five simulated scRNA-seq datasets (TS1, TS2, TS3, and TS4 for tumors; NS for normal tissue). The cell types in TS1, TS2, TS3, and TS4 included cancer cell clone A, cancer cell clone B, ductal cells, fibroblasts, acinar cells, T/NK cells, and red blood cells or their subsets (Supplementary Table S2). In running Bulk2Space, we set the parameters as follows: epoch_num = 3500, *k* = 10, and top_marker_num = 200, and all other parameters as the default values. The simulations were conducted using Python (version 3.8).

### 2.4 Clustering analysis

Clustering analysis was performed using the Non-negative Matrix Factorization (NMF) algorithm implemented via the R package *NMF* (version 0.26.0) [21]. In using NMF for bulk data analysis, we predefined ranks ranging from 2 to 7 and set nrun = 20; in single-cell data analysis, we predefined ranks from 5 to 12 and nrun = 20. The optimal rank or cluster number *k* was selected based on the cophenetic correlation coefficient, when its value had a sharp drop with cluster number *k+*1 compared to that with the cluster number *k*.

### 2.5 Cell-type identification based on pathway enrichment scores by gdGSE

First, we obtained signature gene sets for specific cell types from an original publication (Supplementary Table S3) [18], and transformed the single-cell gene expression matrix into a single-cell signature gene set enrichment matrix using gdGSE. We then employed the NMF algorithm on the gene set enrichment matrix to cluster cells into distinct groups. This approach enabled us to accurately assign each cell to a specific cell type based on its enriched pathways.

### 2.6 Algorithm performance evaluation

To assess the performance of clustering analysis, we utilized several metrics, including the average silhouette coefficient and the cophenetic correlation coefficient. The values of silhouette coefficients range from −1–1, with larger positive values indicating better cluster assignment, low positive values close to 0 indicating a poor cluster assignment, and negative values implying that the samples are likely to be allocated to wrong clusters. In addition, we employed the Adjusted Rand Index (ARI) to quantify the similarity between clustering results and the original clustering annotations. ARI ranges from 0 to 1, with 0 indicating a similarity expected by chance and 1 identical clustering outcomes. Meanwhile, we reported sensitivity, specificity, and balanced accuracy to evaluate the performance of clustering, which are defined as follows:

$\mathrm{Sensivity}=\;\frac{\mathrm{TP}}{\mathrm{TP}+\mathrm{FN}}$

$\mathrm{Specificity}=\;\frac{\mathrm{TN}}{\mathrm{FP}+\mathrm{TN}}$

$\mathrm{Balanced accuracy}=\;\frac{\mathrm{Sensivity}+\mathrm{Specificity}}{2}$，

where TP, FP, TN and FN denote numbers of true positives, false positives, true negatives and false negatives, respectively.

### 2.7 Constructing noise gene expression datasets

To evaluate the robustness of gdGSE, we artificially introduced gene expression noise into true data. We began by generating a gene expression matrix that mirrored the size of the true dataset, using the R function *rnorm* to ensure that the values followed a Gaussian distribution. Subsequently, we created a synthetic gene expression matrix by combining the noise gene expression profiles with the original true data. In using the *rnorm* function, the parameter “mean” was set as 0.01, and the “standard deviation” was set as 0.01, 0.02, 0.03, 0.04, 0.05, and 0.06, respectively.

### 2.8 Computational efficiency benchmarking

To evaluate the computational cost of algorithms, we measured the maximum memory usage during each step of an algorithm and took their maximum value as the algorithm’s maximum memory usage. The memory consumption information was captured by the R function *gc*. For running time evaluation, the time taken for data and package loading, pre- and post-processing steps was excluded. The running time of an algorithm was obtained using the R function *System.time*.

### 2.9 Comparison of gdGSE with other algorithms

We benchmarked gdGSE with nine other GSEA algorithms. These algorithms included three gene set enrichment scoring algorithms used in bulk data analysis: ssGSEA [4], PLAGE [7], and z-score [12], and five algorithms specifically designed for single-cell data: UCell [13], AUCell [5], AddModuleScore [8], and Pagoda2 [9], and singscore [10]. The ssGSEA algorithm is utilized in both bulk and single-cell data. For the benchmarking, we followed their tutorials posted in GitHub and used their default parameter settings. Notably, some algorithms produce gene set enrichment scores that are negative, which are not suitable for NMF. To address this issue, we scaled gene set enrichment scores into the range of [0,1] by the min-max normalization technique. That is, each original gene set enrichment score *x* was scaled as follows:

$$x_{\mathrm{scaled}}=(x-\min\left(x\right))/(\max\left(x\right)-\min\left(x\right)),$$

where $\min\left(x\right)$ and $\max\left(x\right)$ denote the minimum and maximum value of gene set enrichment scores, respectively.

### 2.10 Dimensionality reduction and data visualization

For dimensionality reduction and data visualization in single-cell data analysis, we utilized the R package *Seurat* (version 5.0.2) [22]. We first identified the highly variable genes using the function *FindVariableFeatures* with the default “vst” method, and then performed principal component analysis (PCA) [23] of these genes’ expression profiles in single cells. Using the first 15 principal components, we constructed a shared nearest neighbor (SNN) graph for cells and identified cell clusters with the function *FindClusters*. Finally, we applied the t-distributed Stochastic Neighbor Embedding (t-SNE) method (implemented with the function *RunTSNE*) to visualize cells in low dimensions.

### 2.11 Survival analysis

We used the Kaplan-Meier method [24] to compare survival rates between different classes of cancer patients, and the log-rank test to assess the significance of survival rate differences. Additionally, we employed the univariate Cox proportional hazards model to assess the association between individual parameters and survival prognosis. The parameters having significant correlations with survival (*P* < 0.05) were included in the subsequent multivariable Cox proportional hazard model. The two-tailed Wald test was utilized to assess the significance of association between variates and survival time. All survival analyses were performed with the R package *survival* (version 3.5.7).

### 2.12 Estimating the significance level of gene set enrichment scores by gdGSE

We assessed the significance of a gene set enrichment score (ES) by gdGSE using the permutation test. That is, we permuted the phenotype labels in the original dataset to obtain a new dataset in which we recomputed the ES of the gene set, termed ${\mathrm{ES}}_{\mathrm{perm}}.$ By repeating the permutation experiment *n* time, we obtained *n* ${\mathrm{ES}}_{\mathrm{perm}}.$ The permutation test *P* value indicating the significance of ES by gdGSE was calculated as:

$P=\frac{\mathrm{Number of permutations with}\left|{\mathrm{ES}}_{\mathrm{perm}}\right|\geq \left|{\mathrm{ES}}_{\mathrm{obs}}\right|}{n}$,

where ${\mathrm{ES}}_{\mathrm{obs}}$ represents the ES of the gene set by gdGSE in the original dataset. Here we set *n* = 1000.

### 2.13 Label permutation analysis for false positive calibration in gdGSE

Using rigorous permutation testing, we evaluated the false positive detection performance of the gdGSE algorithm under null conditions. We performed label permutation testing as follows: For the TCGA-CHOL dataset with HALLMARK gene sets, we randomly shuffled sample labels (n = 1000 permutations) to eliminate true biological associations while preserving data structure. In each permutation, we applied gdGSE without multiple testing correction to compute gene set p-values. This process generated a null p-value distribution matrix (gene sets × permutations). Statistical calibration was assessed by sorting all null p-values, binning them into 100 equal-width quantiles (0.00–0.01, 0.01–0.02, etc.), and calculating mean observed versus expected -log10(p-values) per bin. A quantile-quantile (QQ) plot was constructed to visualize agreement between empirical and theoretical null distributions.

## 3 Results

### 3.1 Validation of the gdGSE algorithm in bulk datasets

**Fig. 2:**
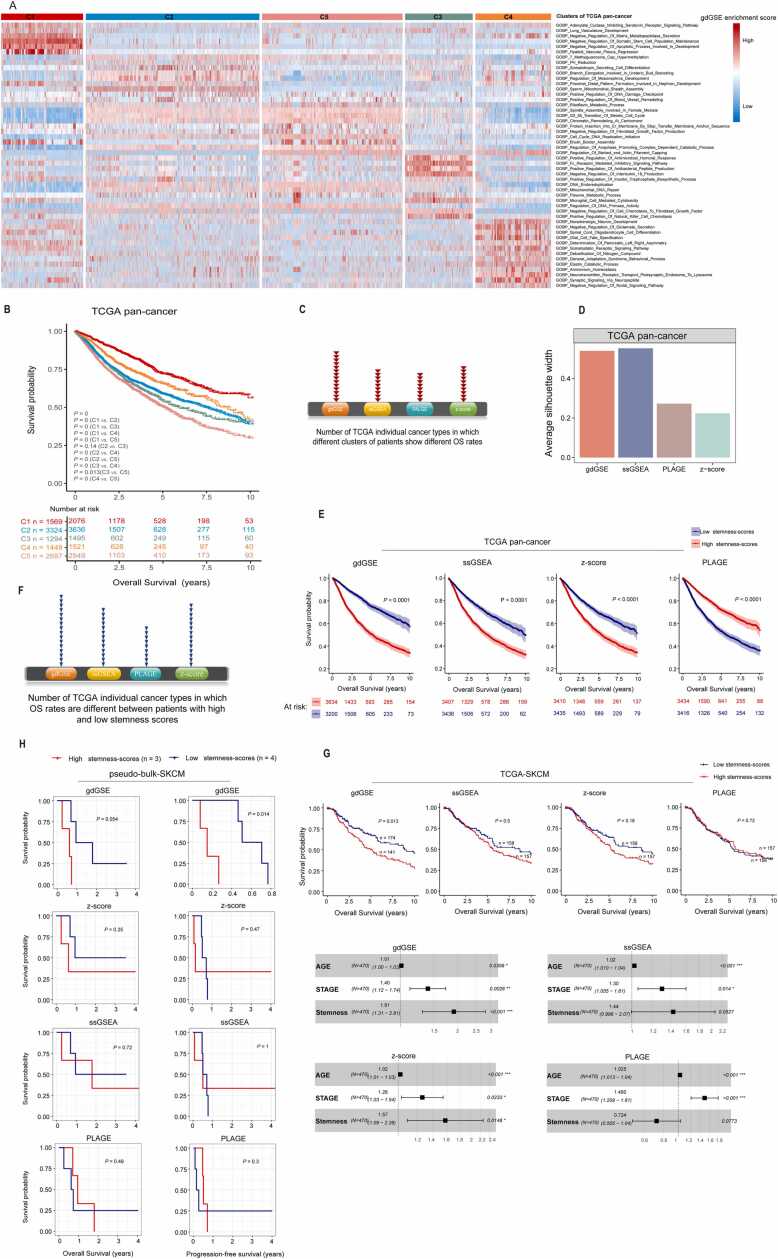
Validation the gdGSE algorithm in bulk datasets. (A) Based on the gdGSE scores of the top 50 pathways which have the highest standard variances of gdGSE scores across all cancer samples, NMF clustering identifies five clusters of the TCGA pan-cancer: C1, C2, C3, C4, and C5. (B) Kaplan-Meier curves displaying that the five clusters of cancer patients have significantly different overall survival (OS) prognosis, with the 10-year OS rate following the pattern: C1 > C4 > C2 > C3 > C5. (C) Number of TCGA cancer types in which different clusters of patients show significantly different OS rates. The clusters of patients were identified using the same procedure as that mentioned in (A). (D) Average silhouette coefficients of clustering results based on the GOBP pathway enrichment scores by different gene set enrichment analysis (GSEA) methods in the TCGA pan-cancer. (E) Kaplan-Meier curves to compare OS time between cancer patients with high stemness signature scores (within upper third) and those with low stemness signature scores (within bottom third) in the TCGA pan-cancer based on different GSEA methods. (F) Number of TCGA cancer types in which OS rates are significantly different between patients with high and low stemness signature scores based on different GSEA methods. (G) Upper panel: Kaplan-Meier curves to compare OS time between cancer patients with high (within upper third) and those with low (within bottom third) stemness signature scores in TCGA-SKCM based on different GSEA methods. The log-rank test *P* values are shown. Lower panel: multivariable Cox proportional hazards regression analysis with the response variable “OS time” and the predictor variables “stemness signature scores”, “tumor stage”, and “age”. (**H**) Kaplan-Meier curves to compare OS and progress-free survival (PFS) between pseudo-bulk-SKCM patients with high (within upper third) and those with low (within bottom third) stemness signature scores. The log-rank test *P* values are shown in (**B**), (**E**), (**G**), and (**H**).

From the MsigDB database [25], we obtained 7750 gene sets representing the Gene Ontology Biological Process (GOBP). We then used gdGSE to calculate these gene sets’ enrichment scores in each cancer sample in the TCGA pan-cancer cohort. The top 50 pathways with the highest standard variances of gdGSE scores across all cancer samples were selected for clustering analysis. This analysis identified five clusters, termed C1, C2, C3, C4, and C5, respectively. C1 showed high enrichment of negative regulation of apoptotic process and stemness cell maintenance pathways; C2 overexpressed pathways of positive regulation of blood vessel remodeling and DNA damage checkpoint; C3 overrepresented immune-related pathways; C4 showed upregulation of neuronal pathways; and C5 displayed high enrichment of cell cycle pathways (Fig. 2A). Survival analysis revealed significant prognostic differences among these subtypes. The 10-year overall survival (OS) rate followed the pattern: C1 > C4 > C2 > C3 > C5 (log-rank test, *P* < 0.0001) (Fig. 2B). This finding aligns with previous reports [3], [26], [27], confirming that overrepresentation of stemness-inhibitory and neuronal pathways is associated with better prognosis in cancer, while upregulation of cell cycle pathways is associated with unfavorable prognosis. Furthermore, we applied gdGSE to 28 individual TCGA cancer types and identified their cancer clusters by NMF. Strikingly, in 14 of the 28 cancer types, the clusters showed markedly distinct OS prognosis (Supplementary Fig. S1A). These results suggest that the pathway enrichment scores by gdGSE can distinguish cancer subtypes with significant prognostic differences. We also used three other GSEA methods (ssGSEA, PLAGE, and z-score) to measure the enrichment levels of GOBP pathways in TCGA cancers and performed clustering analysis based on their measures following the same procedure as above. We found that the cancer clusters identified based on the gene set enrichment scores by ssGSEA, PLAGE, and z-score displayed significantly different OS rates in 9, 8, and 10 cancer types, respectively, compared to 14 cancer types by gdGSE (Fig. 2C). This comparative benchmarking revealed the superior performance of gdGSE versus ssGSEA, PLAGE, and z-score.

To evaluate clustering quality, we computed the average silhouette coefficients of clustering results derived from GOBP pathway enrichment scores generated by gdGSE, ssGSEA, PLAGE, and z-score in the TCGA datasets. In the pan-cancer, gdGSE, ssGSEA, PLAGE, and z-score achieved average silhouette coefficients of 0.54, 0.55, 0.27, and 0.22, respectively. Among 28 individual cancer types, gdGSE obtained the highest average silhouette coefficients in 13 cancer types and the second highest in 10 cancer types (Fig. 2D and Supplementary Fig. S1B). These results indicate that gdGSE-derived pathway activity profiles yield higher-resolution tumor subtyping, likely due to its robustness in handling expression distribution biases.

To assess the clinical relevance of stemness signatures [28], we computed their enrichment scores in TCGA cancers using gdGSE. The cancer patients with high stemness scores (within upper third) exhibited remarkably worse OS than those with low scores (within bottom third) in pan-cancer and in 16 individual cancer types (log-rank test, *P* < 0.05) (Fig. 2E and Supplementary Fig. S1C), validating gdGSE’s ability to quantify biologically meaningful stemness phenotypes. We also evaluated stemness signature scores in TCGA cancers using ssGSEA, PLAGE, and z-score. The stemness signature scores generated by these methods identified survival differences in 13 (ssGSEA), 9 (PLAGE), and 14 (z-score) cancer types, compared to 16 cancer types identified by gdGSE (Fig. 2F). In the pan-cancer cohort, high stemness scores from ssGSEA and z-score correlated with poor survival, whereas scores derived from PLAGE paradoxically associated with improved survival (Fig. 2E).

We further evaluated stemness signature scores by different methods in two SKCM bulk datasets: TCGA-SKCM and pseudo-bulk-SKCM. TCGA-SKCM was a real bulk RNA-seq dataset from TCGA consisting of 469 tumor and 556 normal samples, while pseudo-bulk-SKCM was a synthetic bulk dataset generated by aggregating single-cell transcriptomes from melanoma [16] and a normal skin [29], yielding 10 tumor and 7 normal pseudobulk profiles. In TCGA-SKCM, the patients with high stemness signature scores by gdGSE showed significantly worse OS prognosis than those with low scores (log-rank test, *P* = 0.013) (Fig. 2G). In contrast, the stemness signature scores calculated by ssGSEA, PLAGE, and z-score failed to separate the patients with different OS outcomes (*P* = 0.50, 0.18, and 0.72, respectively). Furthermore, multivariable Cox regression analysis confirmed gdGSE’s stemness scores as an independent adverse prognostic factor after adjusting for age and tumor stage (Fig. 2G). Similarly, in pseudo-bulk-SKCM, the stemness signature scores by gdGSE exhibited prognostic relevance for both OS (*P* = 0.054) and progression-free survival (PFS, *P* = 0.014), whereas conventional methods (ssGSEA, PLAGE, and z-score) demonstrated no significant associations (Fig. 2H).

### 3.2 Benchmarking gdGSE using experimental models with known pathway alterations

**Fig. 3:**
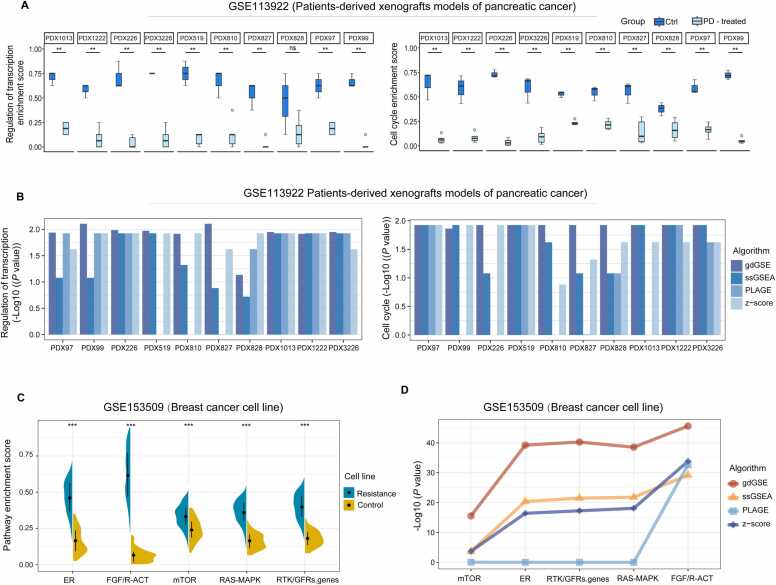
gdGSE benchmarking in models with known pathway alterations. (A) Comparative analysis of transcription and cell cycle signatures were compared control (Ctrl) with palbociclib treated (PD-treated) patients-derived xenograft PDX models of pancreatic cancer. (B) Comparative evaluation of the performance of GSEA (gdGSE, ssGSEA, PLAGE, and z-score) algorithms in distinguishing biologically distinct states (PD-treated VS. Ctrl) across GSE113922 PDX cohort based on transcription and cell cycle signatures enrichment score. (C) Comparing the resistance-associated signature (RTK/GFRs.genes: RTK/growth factor receptors signalling, RAS-MAPK: RAS-MAPK signalling, mTOR: mTOR signalling, ER: ER signalling, FGF/R-ACT: FGF/R-ACT signaling) enrichment scores between the Resistance and Control. (D) To evaluate the performance of GSEA (gdGSE, ssGSEA, PLAGE, and z-score) algorithms in distinguishing biologically distinct states (Resistance vs. Control) across GSE153509 (ER-positive breast cancer cell line) cohort. Resistance: FGFR/FGF resistant group; Control: control group. The one-tailed Mann-Whitney *U* test *P* values are shown. * *P* < 0. 05; * * *P* < 0.01; * ** *P* < 0.001; ^ns^ not significant.

We analyzed the GSE113922 dataset for palbociclib-treated pancreatic cancer PDX models [14], comprising control (Ctrl) and palbociclib treated (PD-treated) groups. The original study demonstrated significant suppression of multiple pathways in PD-treated tumors compared to Ctrl group, including mRNA_splicing, DNA_replication, cell cycle and regulation of transcription pathways. As anticipated, gdGSE pathway enrichment analysis revealed consistent pathway inhibition patterns, with marked downregulation scores observed in these mechanistically related biological pathways in the PD-treated versus Ctrl group (one-tailed Mann-Whitney *U* test, *P* < 0.05) (Fig. 3A and Supplementary Fig. S2A). We then explored whether gdGSE would reveal biological functions consistency with pharmacological expectations. We interrogated an estrogen receptor (ER)-positive breast cancer cell line dataset (GSE153509) [15]. The original publication reported that genetic amplifications of *FGFR1*, *FGFR2* or *FGF3*, and *FGFR2* activating mutations induced treatment resistance through ER pathway reprogramming and concomitant activation of multidrug resistance-associated signaling networks, including signalings of RTK/growth factor receptors (RTK/GFRs.genes), RAS-MAPK signalling (RAS-MAPK), mTOR signalling (mTOR), ER signalling (ER), and FGF/R-ACT signaling (FGF/R-ACT). Using gdGSE, we quantified enrichment levels of these resistance-associated pathways. As expected, these pathways exhibited stronger activation in resistant cell lines compared to controls (one-tailed Mann-Whitney *U* test, *P* < 2.0 × 10^−16^) (Fig. 3 C).

Furthermore, we calculated enrichment scores of the pathways associated with drug treatment by other algorithms. Comparative analyses revealed that enrichment scores derived from gdGSE generally outperformed existing algorithms in discriminating heterogeneous biological phenotypes across PDX and breast cancer cell line cohorts (Fig. 3B&3D and Supplementary Fig. S2A&S2B). These findings collectively demonstrate that gdGSE reliably recapitulates experimentally validated pharmacological responses, establishing its utility as a computational proxy for mechanistic validation.

### 3.3 Cell type identification in single-cell data using gdGSE

**Fig. 4:**
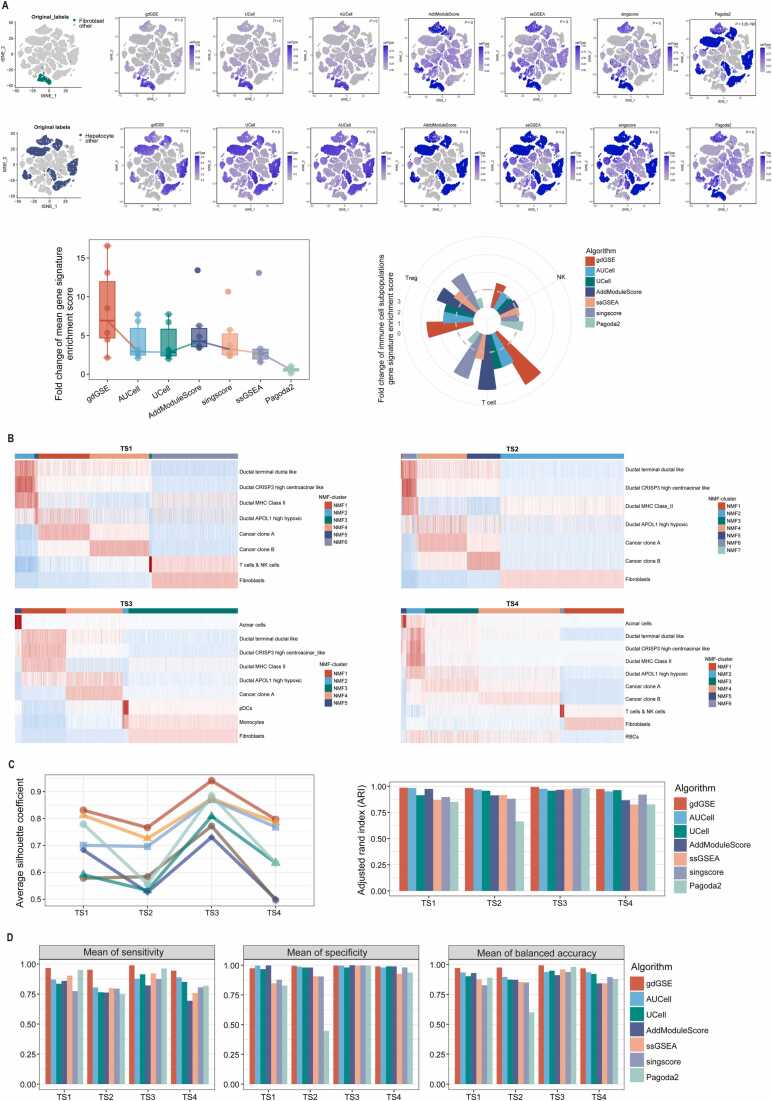
Testing the gdGSE algorithm in single-cell datasets. (A) Upper panel: t-SNE visualization to compare the hepatocyte cells’ and fibroblasts’ gene signatures enrichment scores in the hepatocellular carcinoma (HCC) scRNA-seq dataset (GSE149614) by different GSEA methods. The one-tailed Mann-Whitney *U* test *P* values are shown. Lower panel (left): comparing the fold change of mean gene signature enrichment scores by different algorithms in six major cell types. Lower panel (right): comparing the fold change of gene signature enrichment scores by different algorithms in three immune cell subpopulations. (B) Heatmap showing gene signature enrichment scores by gdGSE in the single cell clusters identified by NMF based on the gdGSE enrichment scores in four simulated PAAD single-cell datasets (TS1, TS2, TS3, and TS4). (C) Comparing the average silhouette coefficient and adjusted rand index (ARI) of the clustering results by NMF based on different GSEA methods in the four simulated PAAD single-cell datasets. (D) Comparing the sensitivity, specificity, and balanced accuracy of cell type identification based on different GSEA methods in four simulated PAAD single-cell datasets.

Cell type identification remains a cornerstone of single-cell transcriptomic analysis [30], where GSEA of cell type-specific signatures is a commonly-used approach. We analyzed both real and simulated single-cell datasets. In the real HCC scRNA-seq dataset GSE149614 [19], which comprises endothelial, fibroblast, hepatocyte, myeloid cells, B cells, and T/NK cells, we used gdGSE to calculate enrichment scores of cell type-specific gene signatures. For each cell type, gdGSE defined two conditions: a specific cell type versus the others, in turn for the specific type of cells. Results showed that enrichment scores of cell type-specific signatures were significantly elevated in their corresponding cell types versus others (one-tailed Mann-Whitney *U* test, *P* < 2.0 × 10^−16^) (Fig. 4A and Supplementary Fig. S3), with mean fold changes exceeding 2. We compared gdGSE’s performance to six widely used single-cell GSEA methods: UCell, AUCell, AddModuleScore, ssGSEA, Pagoda2, and singscore. The gene signature enrichment scores by these algorithms were also significantly higher in a specific cell type than in other cell types, except Pagoda2 that generated an opposite result (Fig. 4A). The fold changes of mean gene signature enrichment scores by these algorithms were mostly lower than those by gdGSE. Furthermore, the original study [19] identified three T/NK subpopulations: Treg, T, and NK cells. Likewise, we evaluated gene signature enrichment scores of these subpopulations by gdGSE. We obtained similar results as previously: the gene signature enrichment scores were higher in a specific cell subpopulation than in other subpopulations (*P* < 2.0 × 10^−16^; fold change = 2.68, 3.48, and 1.44 for Treg, T, and NK cells, respectively) (Fig. 4A). Again, although most of the other algorithms gave higher gene signature enrichment scores in specific cell subpopulations, their fold changes were consistently lower than those by gdGSE. These results imply that the gene signature enrichment scores by gdGSE are likely more outstanding in their specific cell types than those by the other algorithms.

We further evaluated the gdGSE algorithm using four simulated single-cell datasets for PAAD, designated as TS1, TS2, TS3, and TS4. For each dataset, gdGSE defined two conditions: cells from TS1, TS2, TS3, or TS4 and cells from NS (normal tissue), and subsequently calculated gene signature enrichment scores of single cells to obtain the corresponding gene set enrichment matrix. NMF clustering of these matrix identified 6, 7, 5, and 6 cell clusters for TS1, TS2, TS3, and TS4, respectively (Fig. 4B). The average silhouette coefficients of these clustering was 0.83, 0.77, 0.94, and 0.80, respectively, and ARI was 0.99, 0.98, 0.99, and 0.97, respectively (Fig. 4C). To benchmark gdGSE against six GSEA algorithms, we generated their respective gene set enrichment matrices and performed clustering analysis. The results consistently indicated that the average silhouette coefficients and ARIs produced by these algorithms were lower than those achieved by gdGSE (Fig. 4C). We further evaluated the sensitivity, specificity, and balanced accuracy of cell type identification based on gene signature enrichment scores in these simulated single-cell datasets (Supplementary Fig. S4). In all four datasets, the highest mean sensitivity, specificity, and balanced accuracy of cell type identification were achieved by the gdGSE scores-based clustering (Fig. 4D). These results suggest that gdGSE is superior to the other GSEA algorithms for cell type annotation.

### 3.4 Robustness of gdGSE to expression noise in bulk and single-cell data

**Fig. 5:**
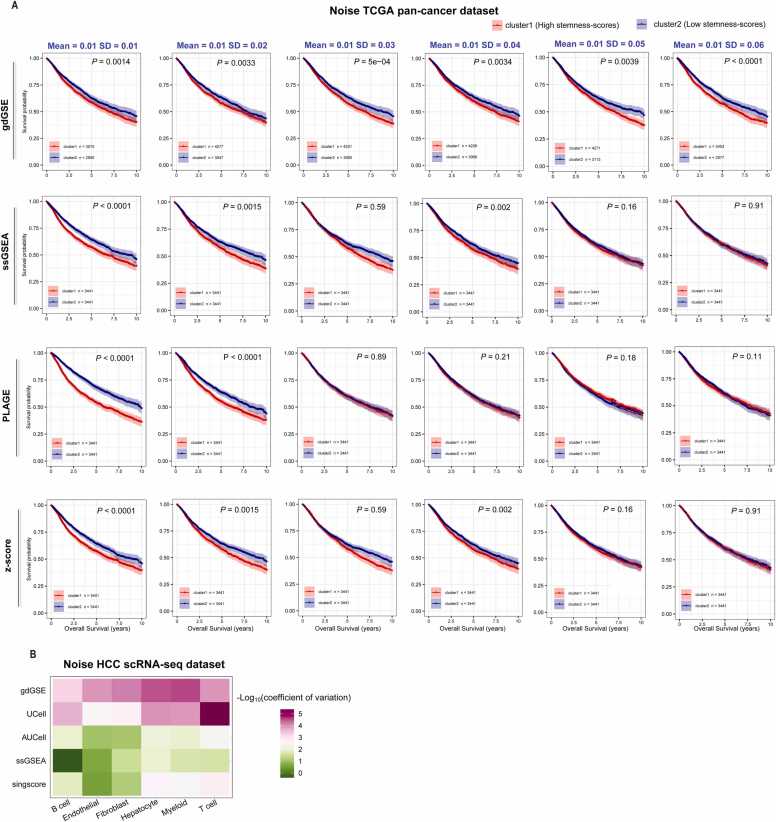
Comparing different GSEA algorithms in noise datasets. (A) Kaplan-Meier curves to compare OS time between cancer patients with high (within upper third) and those with low (within bottom third) stemness signature scores in six noise TCGA pan-cancer datasets based on different GSEA algorithms. The six noise TCGA pan-cancer datasets were generated by introducing Gaussian noise of gene expressions, with mean = 0.01 and standard deviation = 0.01, 0.02, 0.03, 0.04, 0.05, and 0.06, respectively. The log-rank test *P* values are shown. (B) Heatmap for the coefficient of variation (CV) of the fold change of mean gene signature enrichment scores by different GSEA methods across six single-cell noise datasets. The six noise HCC scRNA-seq dataset were generated using the same procedure as mentioned in (A).

To assess robustness of gdGSE, we introduced Gaussian noise (mean = 0.01; standard deviation = 0.01, 0.02, 0.03, 0.04, 0.05, and 0.06) to the TCGA pan-cancer cohort (see Methods and Supplementary Fig. S5A). Despite increasing noise levels, stemness scores derived from gdGSE consistently stratified patients into high- and low-risk groups with significant survival differences (log-rank test, *P* < 0.001) (Fig. 5A). Although in the original TCGA pan-cancer dataset, the stemness scores by other algorithms (ssGSEA, PLAGE, and z-score) also had significant, negative correlations with OS, the correlation became insignificant in the noise datasets when the standard deviation exceeded 0.04 (Fig. 5A). Likewise, we applied identical noise simulations to the HCC scRNA-seq dataset (GSE149614 [19]), analyzing 10,000 randomly sampled tumor cells. We calculated the coefficient of variation (CV), which is the sample standard deviation expressed as a percentage of the mean, for gene signature enrichment scores in individual cell types across the original scRNA-seq dataset and six noise datasets. Notably, gdGSE exhibited lower CV values than UCell, AUCell, ssGSEA, and singscore in all cell types (except a little higher than CV of UCell scores in T/NK cells) (Fig. 5B). These results confirm gdGSE’s reduced sensitivity to technical noise in bulk and single-cell data analyses.

### 3.5 gdGSE exhibits superior computational efficiency in GSEA

**Fig. 6:**
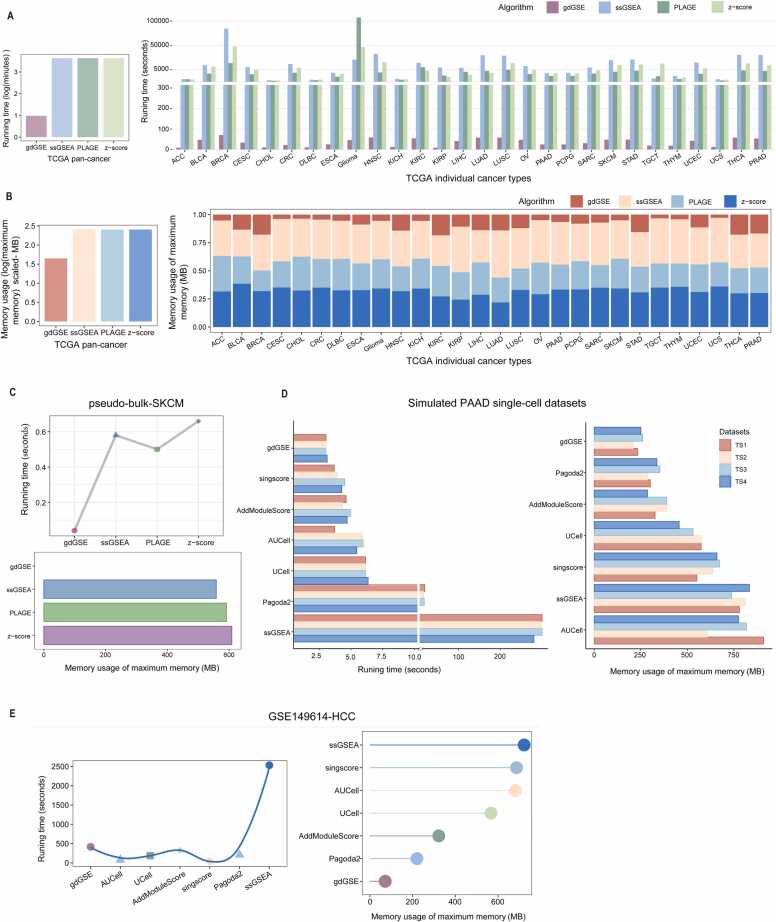
Comparing computational efficiency among different GSEA algorithms. (A, B) Comparing gdGSE with three GSEA algorithms (ssGSEA, PLAGE, and z-score) in running time (A) and maximum memory usage (B) in TCGA pan-cancer and 28 individual cancer types. (C) Comparing gdGSE with three GSEA algorithms (ssGSEA, PLAGE, and z-score) in running time and maximum memory usage in the pseudo-bulk-SKCM dataset. (D, E) Comparing gdGSE with six GSEA algorithms (UCell, AUCell, AddModuleScore, ssGSEA, Pagoda2, and singscore) in running time and maximum memory usage in four simulated PAAD single-cell datasets (TS1, TS2, TS3, and TS4) (D) and TCGA pan-cancer and the real HCC scRNA-seq dataset GSE149614 (E).

It took 10 minutes for gdGSE to compute enrichment scores of 7750 GOBP pathways across 10,323 TCGA tumor samples, while ssGSEA, PLAGE, and z-score required > 72 hours (Fig. 6A); moreover, gdGSE required far less maximum memory than the three algorithms: gdGSE (44.7MB) < ssGSEA (254.3MB) < PLAGE (254.4MB) = z-score (254.4MB) (Fig. 6B). In all 28 TCGA cancer types, gdGSE was the fastest to obtain the results among these algorithms (Fig. 6A), and in most cancer types, it also required the least maximum memory (Fig. 6B). Furthermore, in the pseudobulk RNA-seq dataset pseudo-bulk-SKCM, gdGSE required both the least time and least maximum memory to output the gene set enrichment scores (Fig. 6C). In the simulated PAAD single-cell datasets (TS1, TS2, TS3, and TS4), gdGSE consistently outperformed other algorithms (UCell, AUCell, AddModuleScore, ssGSEA, Pagoda2, and singscore) in terms of both time and memory usage for gene set enrichment evaluation (Fig. 6D). In the real HCC scRNA-seq dataset (GSE149614 [19]), gdGSE took the second-longest time but the least maximum memory to complete the gene signature enrichment evaluation among all seven algorithms (Fig. 6E). Overall, these results demonstrate that gdGSE is a more computationally efficient method for GSEA.

### 3.6 Validation gdGSE algorithm through label permutation analysis

The label permutation analysis reveals excellent agreement between observed and expected p-values under null conditions, as illustrated by the near-identity relationship in the QQ plot across all quantiles (Supplementary Fig. S5C). Minor deviations are observed only at higher quantiles, consistent with the inherent variability expected from finite permutations due to sparse extreme values. The close alignment between observed and expected values, supported by a slope near unity with tight confidence intervals, indicates robust p-value calibration. Importantly, a high proportion of expected p-values align well with their empirically estimated counterparts, demonstrating minimal inflation of false positives.

## 4 Discussion

Here we proposed gdGSE, a novel gene set enrichment scoring method applicable to both bulk and single-cell transcriptomic data. This approach represents the first methodology that evaluates gene set enrichment levels through discretized gene expression values. The data discretization overcomes the discrepancy of different data distribution that leads to the utility of gdGSE in both bulk and single-cell data. In addition, unlike AUCell, UCell, singscore, and ssGSEA, gdGSE measures gene set enrichment levels not relying on the expression ranks of genes. The gene expression rank-based methods have significant limitations: (1) Ranking algorithms are time-consuming; (2) Ranking algorithms compare expression levels of different genes in the same sample but not correcting for their baseline expression differences. By avoidance of these limitations, gdGSE not only outperforms other methods in terms of speed but also circumvents the inherent bias introduced by variations in baseline expression levels across different genes.

The gdGSE framework introduces three critical advancements to GSEA. First, by leveraging condition-aware discretization of gene expression profiles, gdGSE generates pathway activity scores that robustly capture biologically meaningful heterogeneity. Accordingly, the pathway enrichment scores by gdGSE can generate higher clustering quality, as evidenced by better separating tumor patients with significant prognostic differences and more precisely identifying cell subpopulations. The superior clustering fidelity of gdGSE stems from its explicit normalization against reference conditions, which mitigates inter-gene baseline expression biases inherent to rank-based methods. Thus, gdGSE would be advantageous over other algorithms in evaluating pathway enrichment particularly in datasets involving well-characterized conditions, phenotypes or categories. Second, gdGSE exhibits reduced sensitivity to technical noise in transcriptomic studies. In bulk RNA-seq data, it maintained significant survival stratification under Gaussian noise. Similarly, in single-cell data analyses, gdGSE exhibited the lowest CV across noise-injected datasets, ensuring reliable cell type identification even under the interference of technical noise. Third, gdGSE substantially conserves both runtime and computational resources, compared to existing tools, regardless of whether applied to bulk or single-cell data.

Data discretization strategies are mainly based on thresholding, quantile, or clustering methods. Among these methods, thresholding exhibited the best performance in preserving biological signals while also demonstrating greater computational efficiency (Supplementary Fig. S5B). Thus, we adopted the thresholding method for data discretization in designing gdGSE.

Although gdGSE outperforms existing GSEA methods, its reliance on reference condition data (e.g., normal tissue) may limit its applicability in studies lacking control samples. Therefore, to enhance the versatility of gdGSE, future research will focus on extending its discretization logic to accommodate reference-free contexts. By developing methodologies that enable the analysis of gene set enrichment without the necessity for control samples, we aim to broaden the utility of gdGSE in various biological and clinical investigations, ultimately facilitating more inclusive analyses across diverse experimental designs. This adaptation will not only enhance the robustness of the tool but also empower researchers to derive meaningful biological insights in conditions where reference data are scarce or non-existent.

## CRediT authorship contribution statement

**He Mengjiao:** Visualization, Resources. **Qiqi Lu:** Visualization, Validation, Software, Investigation, Formal analysis. **Xiang Yang:** Investigation, Funding acquisition. **Xiaobo Zhang:** Investigation, Funding acquisition. **jiangti Luo:** Writing – original draft, Visualization, Validation, Software, Investigation, Formal analysis, Data curation. **Wang Xiaosheng:** Writing – review & editing, Writing – original draft, Resources, Methodology, Investigation, Conceptualization.

## Funding

This work was supported by the 10.13039/501100002857China Pharmaceutical University (grant number 3150120001 to XW).

## Declaration of Competing Interest

The authors declare that they have no competing interests.

## Data Availability

All datasets used in this study are described in Supplementary Table S1. The R package for implementing the gdGSE algorithm can been freely downloaded from the website: https://github.com/WangX-Lab/gdGSE. The web application of gdGSE can be found at the website: https://cpu-wangx-lab.shinyapps.io/gdGSE/.

## Footnotes

Appendix A Supplementary data associated with this article can be found in the online version at doi:10.1016/j.csbj.2025.04.038.

## References

[1] Subramanian A. (2005). Gene set enrichment analysis: a knowledge-based approach for interpreting genome-wide expression profiles. Proc Natl Acad Sci USA.

[2] Li L., Wang X. (2021). Identification of gastric cancer subtypes based on pathway clustering. npj Precis Oncol.

[3] Lei J.L. (2024). Identifying cancer subtypes based on embryonic and hematopoietic stem cell signatures in pan-cancer. Cell Oncol.

[4] Hänzelmann S., Castelo R., Guinney J. (2013). GSVA: gene set variation analysis for microarray and RNA-Seq data. BMC Bioinforma.

[5] Aibar S. (2017). SCENIC: single-cell regulatory network inference and clustering. Nat Methods.

[6] Santhanam B., Oikonomou P., Tavazoie S. (2023). Systematic assessment of prognostic molecular features across cancers. Cell Genom.

[7] Tomfohr J., Lu J., Kepler T.B. (2005). Pathway level analysis of gene expression using singular value decomposition. BMC Bioinforma.

[8] Tirosh I. (2016). Dissecting Multicell Ecosyst metastatic Melanoma Single-Cell RNA-Seq.

[9] Lake B.B. (2018). Integrative single-cell analysis of transcriptional and epigenetic states in the human adult brain. Nat Biotechnol.

[10] Bhuva D.D., Cursons J., Davis M.J. (2020). Stable gene expression for normalisation and single-sample scoring. Nucleic Acids Res.

[11] Noureen N. (2022). Signature-scoring methods developed for bulk samples are not adequate for cancer single-cell RNA sequencing data. eLife.

[12] Lee E. (2008). Inferring pathway activity toward precise disease classification. PLOS Comput Biol.

[13] Andreatta M., Carmona S.J. (2021). UCell: Robust and scalable single-cell gene signature scoring. Comput Struct Biotechnol J.

[14] Knudsen E.S. (2019). Cell cycle plasticity driven by MTOR signaling: integral resistance to CDK4/6 inhibition in patient-derived models of pancreatic cancer. Oncogene.

[15] Mao P. (2020). Acquired FGFR and FGF Alterations Confer Resistance to Estrogen Receptor (ER) Targeted Therapy in ER+ Metastatic Breast Cancer. Clin Cancer Res.

[16] Barras D. (2024). Response Tumor-infiltrating Lymph Adopt Ther Is Assoc preexisting CD8+ T-myeloid Cell Netw Melanoma.

[17] Merleev A. (2022). Proprotein convertase subtilisin/kexin type 9 is a psoriasis-susceptibility locus that is negatively related to IL36G. JCI Insight.

[18] Moncada R. (2020). Integrating microarray-based spatial transcriptomics and single-cell RNA-seq reveals tissue architecture in pancreatic ductal adenocarcinomas. Nat Biotechnol.

[19] Lu Y. (2022). A single-cell atlas of the multicellular ecosystem of primary and metastatic hepatocellular carcinoma. Nat Commun.

[20] Liao J. (2022). De novo analysis of bulk RNA-seq data at spatially resolved single-cell resolution. Nat Commun.

[21] Gaujoux R., Seoighe C. (2010). A flexible R package for nonnegative matrix factorization. BMC Bioinforma.

[22] Hao Y. (2024). Dictionary learning for integrative, multimodal and scalable single-cell analysis. Nat Biotechnol.

[23] Jolliffe I., Lovric M. (2011). in *International Encyclopedia of Statistical Science*.

[24] Bland J.M., Altman D.G. (1998). Survival probabilities (the Kaplan-Meier method). BMJ.

[25] Liberzon A. (2015). The Molecular Signatures Database (MSigDB) hallmark gene set collection. Cell Syst.

[26] Feng Q., Song D., Wang X. (2021). Pan-cancer analysis reveals that neurotrophin signaling correlates positively with anti-tumor immunity, clinical outcomes, and response to targeted therapies and immunotherapies in cancer. Life Sci.

[27] Cavalu S. (2024). Cell cycle machinery in oncology: a comprehensive review of therapeutic targets. Faseb J.

[28] Miranda A. (2019). Cancer stemness, intratumoral heterogeneity, and immune response across cancers.

[29] van Straalen K.R. (2023). Single cell sequencing reveals Hippo signaling as a driver of fibrosis in hidradenitis suppurativa. J Clin Investig.

[30] Butler A. (2018). Integrating single-cell transcriptomic data across different conditions, technologies, and species. Nat Biotechnol.

