# Supplementary figures and images for: gdGSE: An algorithm to evaluate pathway enrichment by discretizing gene expression values
Source: Comput Struct Biotechnol J. 2025 May 1;27:1772–83. doi: 10.1016/j.csbj.2025.04.038 (PMC12127574; doi:10.1016/j.csbj.2025.04.038)

Supplementary Fig 1

A

gdGSE

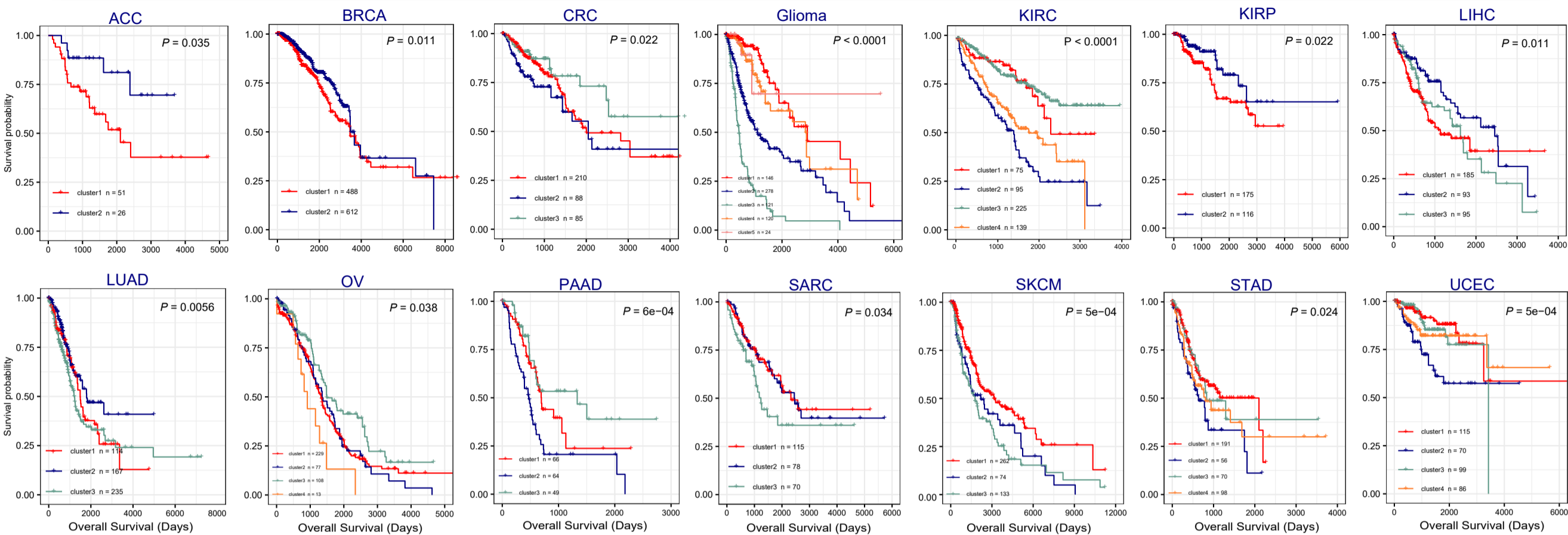

B

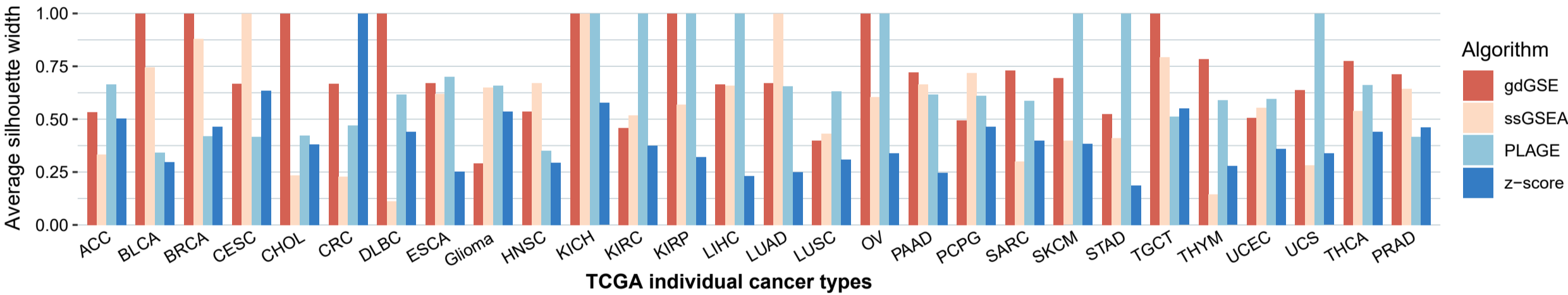

C

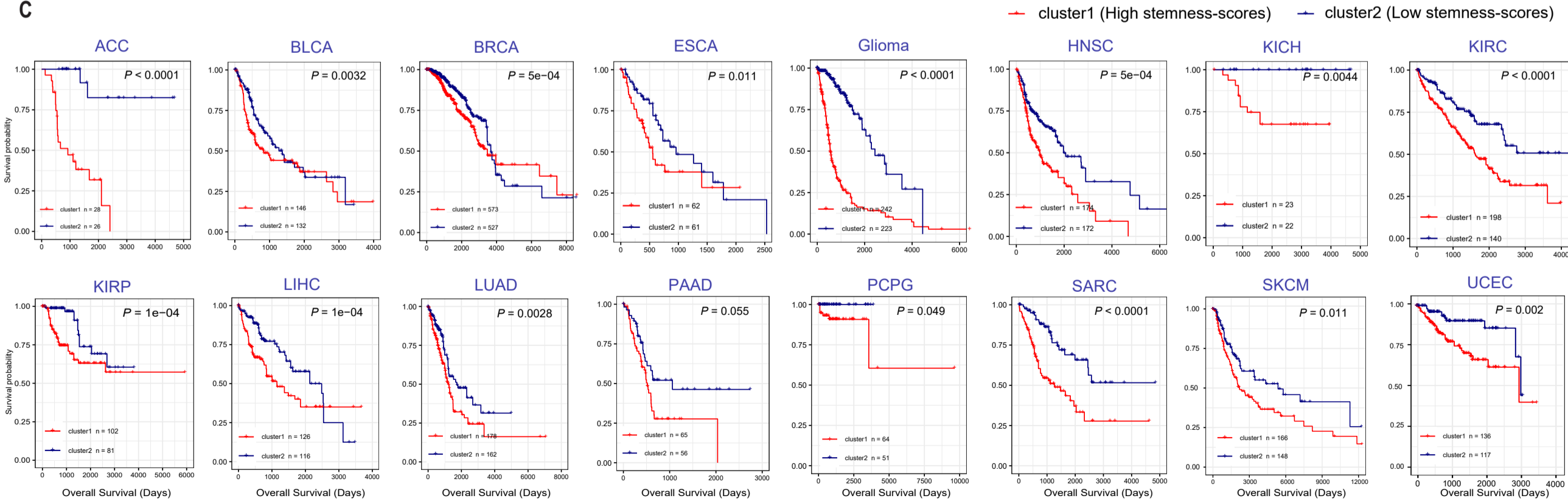

Supplement: Supplementary file 2 — Supplementary material [file mmc2.pdf]

Supplementary Fig 2

A

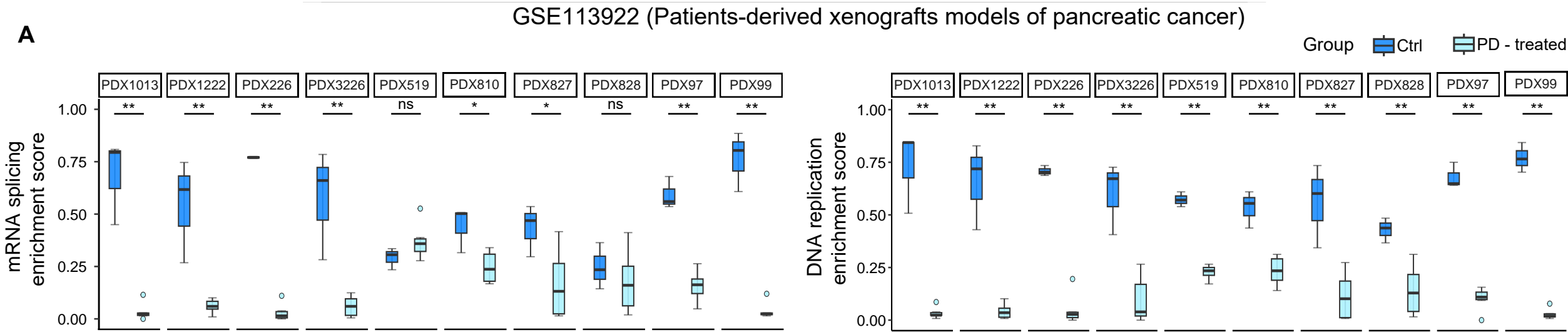

B

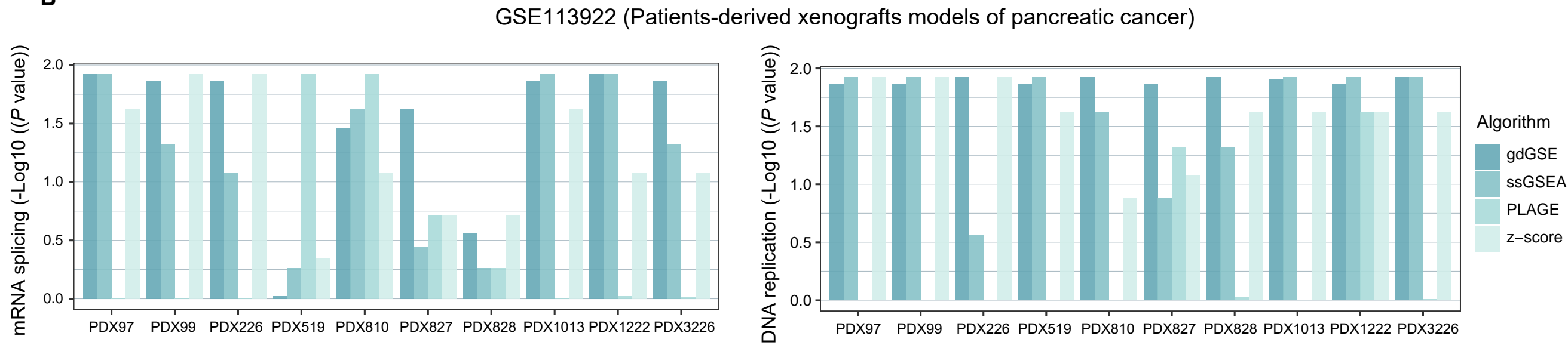

Supplement: Supplementary file 3 — Supplementary material [file mmc3.pdf]

Supplementary Fig. S3

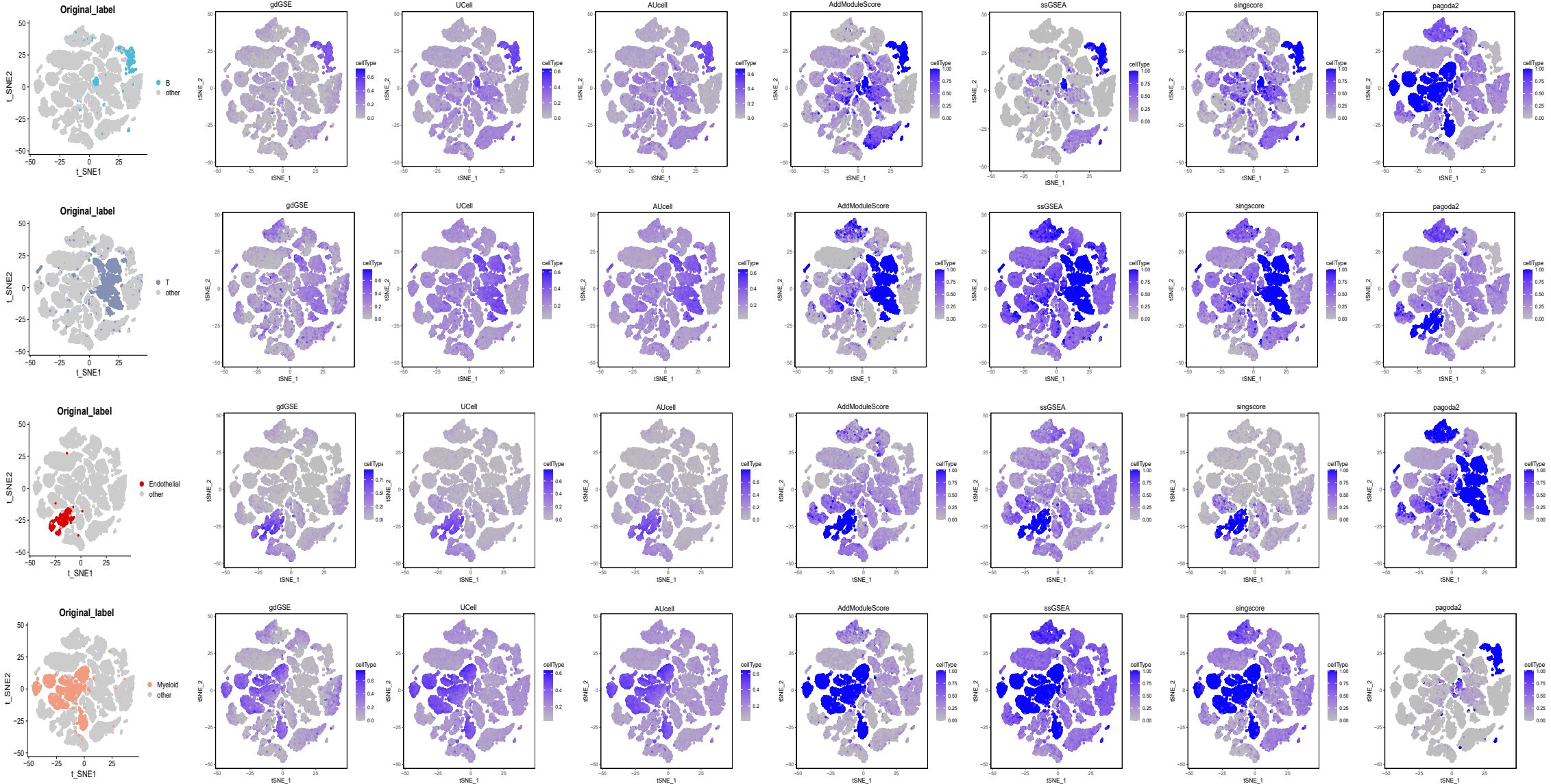

Supplement: Supplementary file 4 — Supplementary material [file mmc4.pdf]

Supplementary Fig 5

A

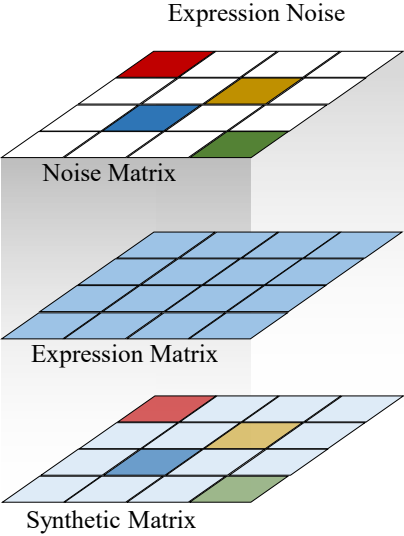

B

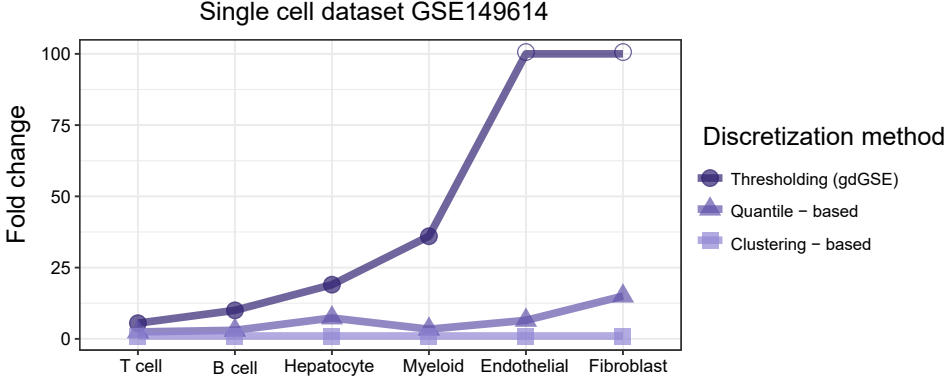

C

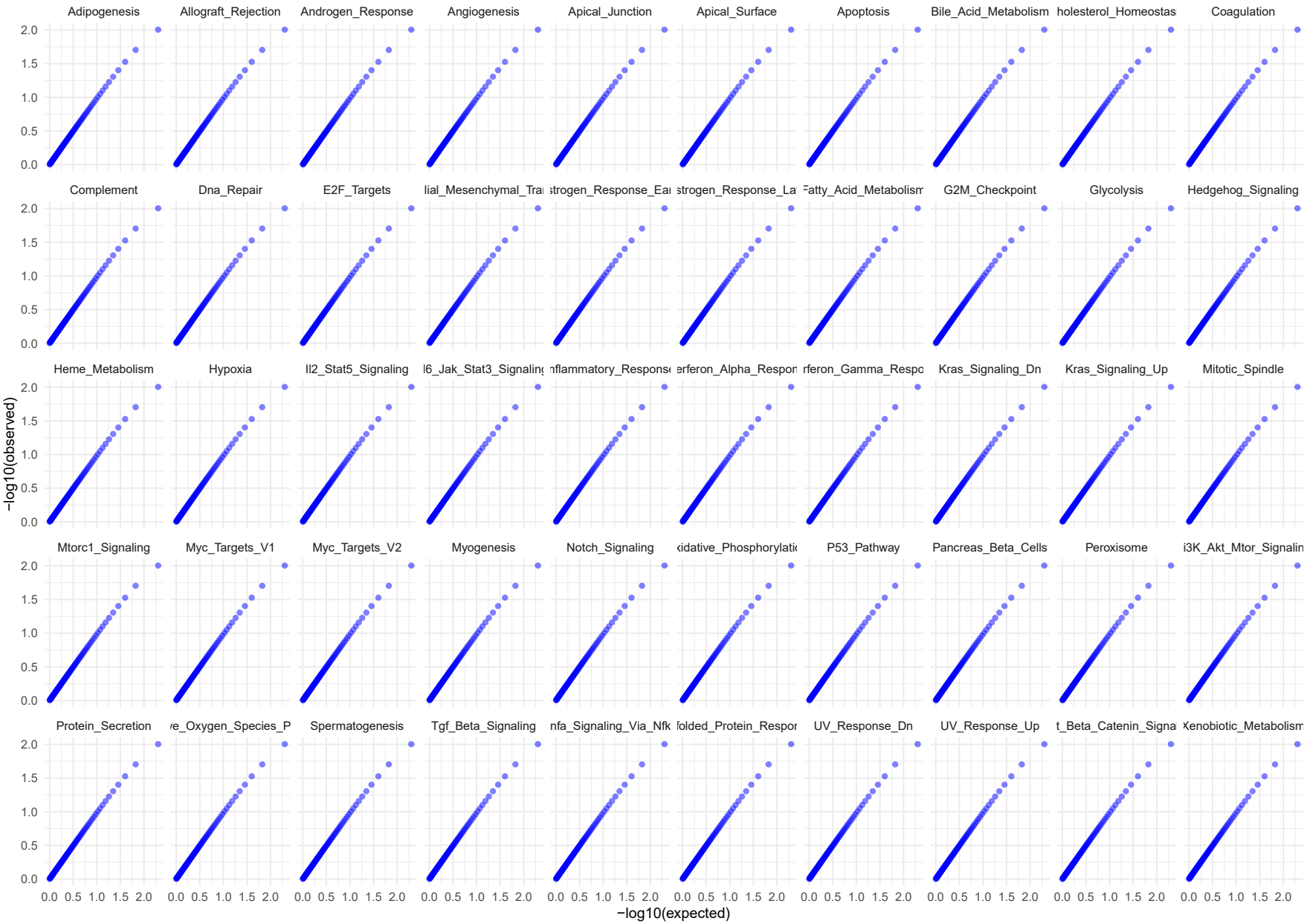

Supplement: Supplementary file 5 — Supplementary material [file mmc5.pdf]

Supplementary Fig 4

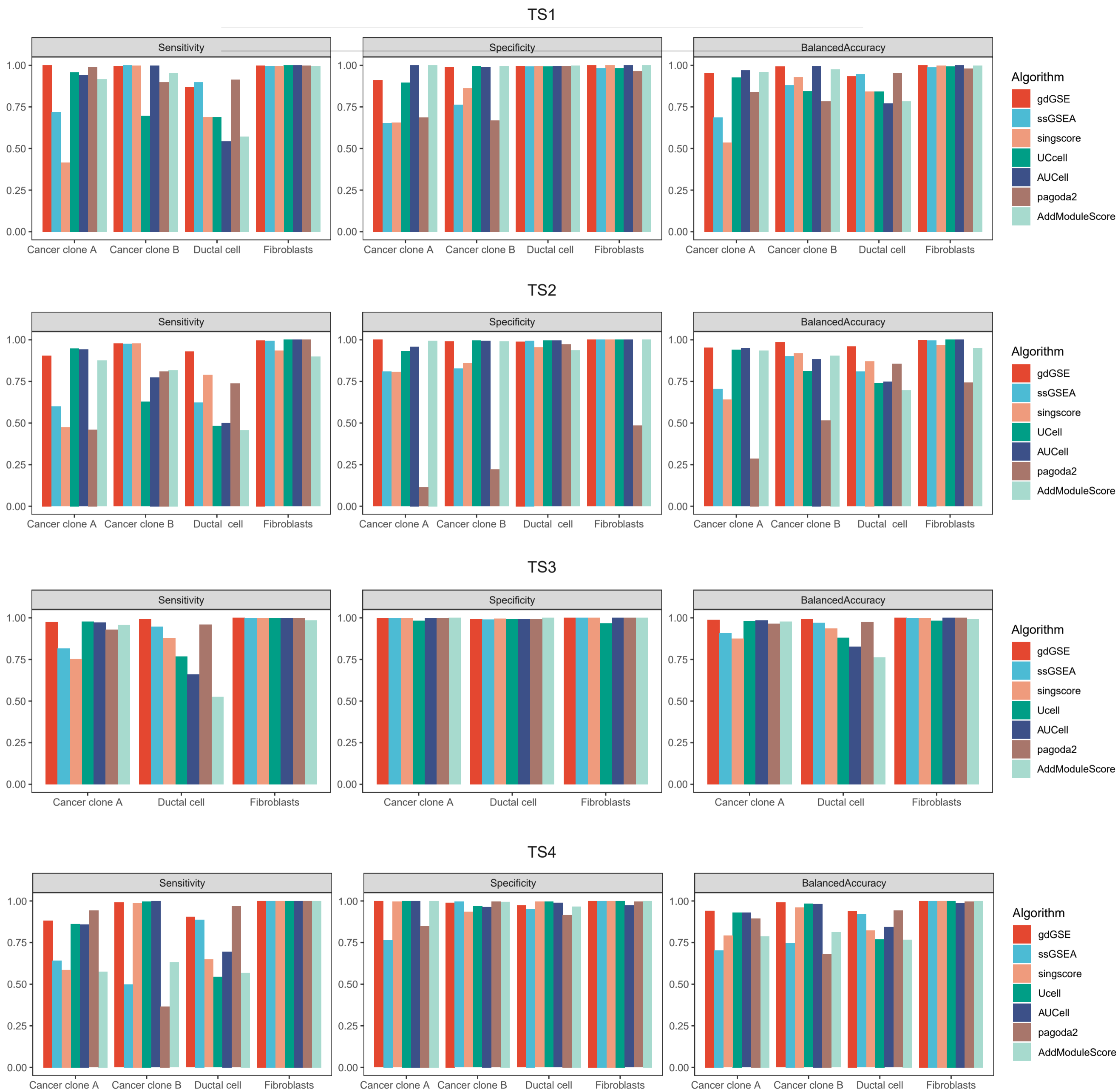

Supplement: Supplementary file 6 — Supplementary material [file mmc6.pdf]
